# T-Cell Replete Myeloablative Haploidentical Bone Marrow Transplantation Is an Effective Option for Pediatric and Young Adult Patients With High-Risk Hematologic Malignancies

**DOI:** 10.3389/fped.2020.00282

**Published:** 2020-06-09

**Authors:** Emmanuel Katsanis, Lauren N. Sapp, Susie Cienfuegos Reid, Naresh Reddivalla, Baldassarre Stea

**Affiliations:** ^1^Department of Pediatrics, University of Arizona, Tucson, AZ, United States; ^2^Department of Immunobiology, University of Arizona, Tucson, AZ, United States; ^3^Department of Medicine, University of Arizona, Tucson, AZ, United States; ^4^Department of Pathology, University of Arizona, Tucson, AZ, United States; ^5^The University of Arizona Cancer Center, Tucson, AZ, United States; ^6^Banner University Medical Center, Tucson, AZ, United States; ^7^Banner Cardon Children's Medical Center, Mesa, AZ, United States; ^8^Department of Radiation Oncology, University of Arizona, Tucson, AZ, United States

**Keywords:** myeloablative, T-replete, haploidentical BMT, pediatric, GvHD

## Abstract

Twenty-one pediatric and young adult patients (1.1–24.7 years) with hematologic malignancies underwent myeloablative T-cell replete haploidentical bone marrow transplant (haplo-BMT) between October 2015 to December 2019. Fifty-seven percent of the patients were ethnic or racial minorities. Thirteen patients had B-cell precursor acute lymphoblastic leukemia (B-ALL) with 10 receiving 1,200 cGy fractionated total body irradiation with fludarabine while the remaining 11 patients had targeted dose-busulfan, fludarabine, melphalan conditioning. Graft-vs.-host disease (GvHD) prophylaxis consisted of post-transplant cyclophosphamide (15 patients) or cyclophosphamide and bendamustine (six patients), with all patients receiving tacrolimus and mycophenolate mofetil. Twelve patients were in first or second remission at time of transplant with five in >2nd remission and four with measurable disease. Three patients had failed prior transplants and three CAR-T cell therapies. Only one patient developed primary graft failure but engrafted promptly after a second conditioned T-replete peripheral blood stem cell transplant from the same donor. An absolute neutrophil count of 0.5 × 10^9^/L was achieved at a median time of 16 days post-BMT while platelet engraftment occurred at a median of 30 days. The cumulative incidence of grades III to IV acute GvHD and chronic GvHD was 15.2 and 18.1%, respectively. With a median follow-up of 25.1 months the relapse rate is 17.6% with an overall survival of 84.0% and a progression-free survival of 74.3%. The chronic graft-vs.-host-free relapse-free survival (CRFS) is 58.5% while acute and chronic graft-vs.-host-free relapse-free survival (GRFS) is 50.1%. Myeloablative conditioned T-replete haploidentical BMT is a viable alternative to matched unrelated transplantation for children and young adults with high-risk hematologic malignancies.

## Introduction

There has been a global resurgence in the use of haploidentical hematopoietic cell transplantation (haplo-HCT). One of the factors that has contributed to this increase has been the ability to use T-replete stem cell grafts whether bone marrow (BM) or peripheral blood (PBSC) followed by post-transplant cyclophosphamide (PT-CY) (1–5). While there have been countless adult reports using haplo-HCT with PT-CY for hematologic malignancies, application of this approach in pediatric patients has lagged behind. Potential contributing factors may have included the comfort level and familiarity of using matched unrelated donor (MUD) or umbilical cord blood (UCB) by pediatric transplant physicians, the absence of haplo-HCT as a transplant option in many cooperative group trials, and the relative lack of pediatric haplo-HCT publications demonstrating comparable efficacy to MUD transplantation. Moreover, the most widely published conditioning regimen used with PT-CY has been a reduced intensity conditioning (RIC) consisting of low dose cyclophosphamide, fludarabine and 200 cGy total body irradiation (TBI) developed at Johns Hopkins, which is not ideal for high-risk pediatric acute leukemias as relapses have been reported in more than half of patients (Table 1) (8).

**Table 1:** Pediatric T-cell replete haploidentical hematopoietic cell transplant studies with PT-CY in patients with hematologic malignancies.

	***n***	**Single or multi-institution**	**Age**	**Disease**	**Disease status%**	**Regimen%**	**Graft**	**GvHD prophy**	**Engraft%**	**aGvHD III-IV%**	**cGvHD%**	**NRM%**	**Relapse%**	**OS %**	**PFS%**	**F/U mo**
Berger et al. (6)	33	M-#5 Italy	1–21	ALL 45% AML 21% OL 3% MDS 12% CML 3% HD-NHL 5%	24 CR1 24% 30 CR2 30% 15 >CR2 15% NR 30%	RIC 57% CY-FLU-200cGy MAC 43% BU-FLU-TT 1,200cGy-FLU	BM	PT-CY Tacro MMF	97	3	4	9	24	72 @1yr	61 @1 yr	12
Jaiswal et al. (7)	20	S India	2–20	ALL 35% AML 65%	NR 100%	MAC BU-FLU-MEL	PBSC	PT-CY CSA MMF	100	20	5	20	26	64 @2yr	59 @2 yr	22
Klein et al. (8)	40	S Baltimore MD, US	1–25	ALL 23% AML 23% MDS 13% AUL 2% CML 2% HD-NHL 38%	CR 43 % NR 57%	RIC CY-FLU-200cGy	BM	PT-CY Tacro MMF	94	13	24 7*	13	52	56 @1yr	43 @1 yr	20
Katsanis et al. (9)	13	S Tucson AZ, US	4–26	ALL 54% AML 15% AUL 7% CML 7% HD-NHL 15%	CR1 23% CR2 46% >CR2 8% NR 23% 2nd BMT 15% failed CAR-T 7%	MAC BU-FLU-MEL 1,200cGy-FLU	BM	PT-CY PT-CY/BEN Tacro MMF	100	0	29 19*	0	0	100 @1yr	100 @1 yr	15
Hong et al. (10)	22	S South Korea	1–20	ALL 50% AML 32% AUL 14% HD 14%	CR1 73% >CR2 27%	MAC BU-FLU-CY	PBSC	PT-CY Tacro MMF	100	6	9*	0	21	82 @2yr	78 @2 yr	26
Symons et al. (11) Abstract	32	M-#9 US-Canada	1–23	ALL 41% AML 41% AUL 3% MDS 15%	CR1 47% CR2 34% 2nd BMT 3%	MAC BU-CY 1,200cGy-CY	BM	PT-CY Tacro MMF	84	0	4*	0	32	77 @1yr	68 @1 yr	39
Katsanis Current	21	S Tucson AZ, US	1–24	ALL 62% AML 14% AUL 5% CML 5% HD-NHL 14%	CR1 19% CR2 38% >CR2 24% NR 19% 2nd BMT 14% failed CAR-T 14%	MAC BU-FLU-MEL 1,200cGy-FLU	BM	PT-CY PT-CY/BEN Tacro MMF	95	15	18 12*	9	17	91 @1yr 84 @2 yr	86 @1 yr 74 @2 yr	25

*n, number; GvHD, graft-vs.-host disease; a, acute; c, chronic; NRM, non-relapse mortality; OS, overall survival; PFS, progression-free survival; F/U, follow-up; M, multi-institutional; #, number of institutions; S, single institution*;

ALL, acute lymphoblastic leukemia; AML, acute myeloid leukemia; OL, other leukemia; MDS, myelodysplastic syndrome; CML, chronic myeloid leukemia; HD, Hodgkin's disease; NHL, non-Hodgkin lymphoma; AUL, acute undifferentiated leukemia; CR, complete remission; NR, not in remission; PT-CY, post-transplant cyclophosphamide; PT-CY/BEN, post-transplant cyclophosphamide/bendamustine; MAC, myeloablative conditioning; RIC, reduced intensity conditioning; *, extensive cGvHD.

Only a handful of pediatric studies using T-cell replete grafts with myeloablative conditioning (MAC) followed by PT-CY have been published (Table 1). An Italian group described their pediatric haploidentical bone marrow transplantation (haplo-BMT) experience from five centers, with 14 patients (43%) receiving MAC and the remaining RIC, all followed by PT-CY (6). A team from India reported on the use of PT-CY following a chemotherapy based MAC regimen and unmanipulated PBSC transplant in 20 pediatric leukemia patients in India (7). More recently, a South Korean group reported on 22 pediatric patients with hematologic malignancies also undergoing PBSC transplant using a chemotherapy-based MAC and PT-CY (10). We have previously published our initial experience with MAC haplo-BMT using TBI-fludarabine primarily for B-cell precursor acute lymphoblastic leukemia (B-ALL) and busulfan, fludarabine, melphalan for the other hematologic malignancies (9). Herein, we update our results with MAC T-cell replete haplo-BMT, making this the largest single institution pediatric report in North America. Our experience confirms that T-cell replete haplo-BMT utilizing MAC is a safe and effective HCT modality for pediatric and young adults with hematologic malignancies and should be readily offered to HCT candidates without a matched sibling donor.

## Methods

### Patients

Twenty-one pediatric and young adult patients (1.1–24.7 years) with hematologic malignancies underwent myeloablative T-cell replete haplo-BMT on the pediatric hematopoietic cell therapy and transplant (HCTT) service at Banner University Medical Center from October 2015 to December 2019. University of Arizona institutional review board approval was obtained to review and report our findings. Eligible patients for haplo-BMT were those who had no matched related donor or a readily available MUD, met organ criteria allowing for MAC and had no evidence of active untreated infection.

### Conditioning Regimens

Ten patients were conditioned with fractionated TBI of 200 cGy BID given on days −8, −7, and −6 (1,200 cGy total dose with lungs shielded to 900 cGy by custom cerrobend blocking), followed by fludarabine (FLU) 30 mg/m^2^ on days −5, −4, −3, and −2 (2, 9). Eleven patients received busulfan (BU) at 0.8 mg/kg IV every 6 h for a total of 12 doses (days −8 to −6), targeting an average area under the curve (AUC) of 1,000–1,100 μMol/min for the duration of the course. BU pharmacokinetics of the first dose were performed at the Seattle Cancer Care Alliance laboratory. The seventh and remaining doses were modified to achieve the average exposure of 1,000–1,100 μMol/min. BU was followed by FLU 30 mg/m^2^ on days −5, −4, −3, and −2 and melphalan (MEL) 100 mg/m^2^ on day −2 (9, 12).

### Graft-vs.-Host Disease (GvHD) Prophylaxis

Fifteen patients received PT-CY 50 mg/kg on days +3 and +4. Six patients that were part of an IRB–approved phase I single institution clinical trial through the University of Arizona Cancer Center (NCT02996773) received PT-CY 50 mg/kg on days +3 and post-transplant bendamustine (PT-BEN) with or without PT-CY on day +4. One patient (cohort 1) received 40 mg/kg PT-CY on day +4, immediately followed by PT-BEN 20 mg/m^2^. Two patients (cohort 2) were treated PT-CY 20 mg/kg followed by PT-BEN 60 mg/m^2^ while three patients (cohort 3) received only PT-BEN 90 mg/m^2^ on day +4. All patients were started on mycophenolate mofetil on day +5 until day +28 and Tacrolimus from day +5. In the absence of GvHD, tacrolimus was weaned starting day +70 to +90 and discontinued by day +120 to 180. GvHD was graded according to the consensus criteria for grading acute and chronic GVHD (13, 14).

### Supportive Care

Antifungal prophylaxis with voriconazole was administered in all patients. Patients received i.v. pentamidine for Pneumocystis jirovecii and acyclovir for herpes simplex and varicella virus prophylaxis. Bi-weekly polymerase chain reaction (PCR) monitoring for cytomegalovirus (CMV) and weekly for adenovirus, Epstein-Barr virus (EBV) and human herpes virus-6 (HHV-6) were performed until discharge from hospital and subsequently at least every other week during first 100 days. All patients were transplanted in HEPA filtered rooms on a HEPA filtered unit and encouraged to walk laps on the unit daily.

### Donor Selection

Donors were first degree relatives who were HLA-haploidentical based on high-resolution typing at HLA-A, -B, -Cw, -DRB1, and -DQB1. Fourteen of the donors were five of 10 antigen matches, five were six of 10 and two 7/10 (Table 2). None of the patients had anti-donor HLA antibodies. There were eight major, and four minor ABO incompatibilities that required donor red blood cell (RBC) reduction using Hespan® (6% hetastarch in 0.9% sodium chloride injection) for RBC sedimentation or plasma reduction, respectively (15).

**Table 2:** Patient, disease and transplant characteristics.

**Age**, median yr, (range)	16.8 (1.1–24.7)
**Male** gender, *n* (%)	14 (66.7)
**Race/Ethnicity, *n* (%)**	
White Hispanic	10 (47.6)
Native American	1 (4.8)
African American	1 (4.8)
White	9 (42.9)
**Diagnosis, *n* (%)**	
B-ALL	13 (61.9)
AML	3 (14.3)
AUL	1 (4.8)
CML	1 (4.8)
NHL	2 (9.5)
HD	1 (4.8)
**Pretransplant Status, *n* (%)**	
CR1	4 (19)
CR2	8 (38.1)
>CR2	5 (23.8)
Other	4 (19)
Prior BMT	3 (14.3)
Failed prior CAR-T	3 (14.3)
**Disease risk index, *n* (%)**	
Low	1 (4.8)
Intermediate	19 (90.4)
High	1 (4.8)
**Lansky/Karnofsky**, median (range)	90 (50–100)
**HCT Comorbidity index**, median (range)	0 (0–7)
**Conditioning, *n* (%)**	
TBI-FLU	10 (47.6)
BU-FLU-MEL	11 (52.4)
**GvHD prophylaxis**	
PT-CY, Tacro, MMF	15 (71.4)
PT-CY/BEN, Tacro, MMF	6 (28.6)
**Graft composition median (range)**	
CD34+ × 10^6^/kg	4.05 (1.5–7.5)
**RBC incompatibility, *n* (%)**	
None	9 (42.9)
Major	8 (38.1)
Minor	4 (19.0)
**Donor age**, median yr, (range)	34.7 (16.3–47.7)
**Donors of male recipients, *n* (%)**	
Mother	6 (42.9)
Father	4 (28.6)
Brother	4 (28.6)
**Donors of female recipients, *n* (%)**	
Mother	2 (28.6)
Father	2 (28.6)
Sister	2 (28.6)
Brother	1 (14.3)
**Donor Match, *n* (%)**	
5/10	14 (66.7)
6/10	5 (23.8)
7/10	2 (9.5)

*ALL, acute lymphoblastic leukemia; AML, acute myeloid leukemia; AUL, acute undifferentiated leukemia; CML, chronic myeloid leukemia; NHL, non-Hodgkin lymphoma; HD, Hodgkin's dsease; CR, complete remission; TBI, total body irradiation; FLU, fludarabine; BU, busulfan; MEL, melphalan; CY, cyclophosphamide; BEN, bendamustine; Tacro, Tacrolimus; MMF, mycophenolate mofetil*.

### Engraftment and Donor Chimerism Monitoring

Granulocyte-colony stimulating factor (G-CSF) was started on day +5 at 5 μg/kg/day until an absolute neutrophil count (ANC) of 2.5 × 10^9^/L was achieved for three consecutive days. Day of myeloid engraftment was defined as the first of three consecutive days with an ANC of 0.5 × 10^9^/L. Day of platelet engraftment was considered the first of three consecutive days with platelet counts of >20 × 10^9^/L with no platelet transfusions administered in the previous 7 days. Donor chimerism was evaluated on days +28, +100, +180, and +365 by short tandem repeats (STRs) on peripheral blood or bone marrow. Engraftment testing was performed using labeled primers to PCR-amplify STR polymorphic DNA markers followed by capillary electrophoresis to distinguish between the DNA contributed by the recipient vs. the donor, and estimate the percentage of the contribution. The Promega GenePrint 24 System which includes 24 polymorphic markers was used (Promega Corporation, Madison, WI) (16).

### Statistical Analysis

Time to event endpoints were estimated using the Kaplan-Meier method. The association between the number of CD34^+^ × 10^6^/kg cells infused with time to neutrophil or platelet engraftment was estimated using linear regression analysis.

## Results

### Patient, Disease, and Transplant Characteristics

Twenty-one patients with hematologic malignancies underwent T-replete haplo-BMT following myeloablative conditioning. The clinical characteristics of the patients are outlined in Table 2. The median age at transplant was 16.8 years (1.1–24.7 years). Two thirds of patients were male. Ethnic and/or racial minorities constituted 57% of all patients, the majority of whom (48%) were Hispanic. Sixty-two percent of the patients had B-ALL. All 13 patients with B-ALL were minimal residual disease (MRD) negative by flow cytometry prior to initiation of conditioning. Only one patient was in first complete remission (CR1), who had failed two chemotherapy induction regimens and achieved CR1 only after blinatumomab, while seven were in CR2 and five in third or greater remission. One B-ALL patient had haplo-BMT following a prior MUD BMT, one following failure of chimeric antigen receptor (CAR)-T therapy and one following relapse after unrelated cord blood transplant and failure of multiple CAR-T cell therapies (17). Cytogenetics were unfavorable in nine of the 13 patients with B-ALL including two with 9:22 translocation, two with 11q23.3 translocation, three with intrachromosomal amplification of chromosome 21 (iAMP21) with RUNX1 amplification and two with complex abnormalities. Two patients had *de novo* acute myeloid leukemia (AML) one of which had 11q23/MLL-rearranged AML, another patient developed a secondary AML eight months following completion of chemotherapy for osteogenic sarcoma and also was t(9,11),11q23 positive (12) and one had acute undifferentiated leukemia. All of these patients were in morphologic remission, three in CR1 and one AML in CR2. One patient had chronic myelogenous leukemia (CML) in chronic phase and had failed tyrosine kinase inhibitor therapy, having developed T315I kinase domain mutation. The other three patients had relapsed/refractory Hodgkin's (HD) or non-Hodgkin lymphoma (NHL). One patient with HD was in partial remission following an autologous PBSC transplant. One patient with refractory diffuse large B-cell lymphoma (DLBCL) had never achieved complete remission having failed multiple lines of therapy including CAR-T cells and was in partial remission at the time of transplant as was a patient with anaplastic large cell lymphoma.

Ten of the B-ALL (77%) patients were conditioned with fractionated TBI followed by fludarabine (TBI-FLU) while the other three (one infant with B-ALL, one who had a prior MUD BMT with TBI, and one with poor performance status) received BU-FLU-MEL. All other patients with non-lymphoid leukemias and those with lymphomas received BU-FLU-MEL.

### Engraftment and Chimerism

**Figure 1:**
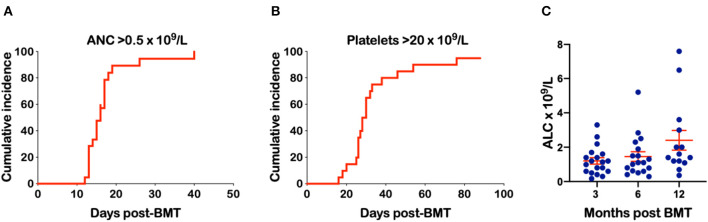
Engraftment of neutrophils and platelets and absolute lymphocyte count (ALC) over time. **(A)** Time to an ANC of 0.5 × 10^9^/L. **(B)** Time to a platelet count of 20 × 10^9^/L **(C)** ALC at 3, 6, and 12 months following haplo-BMT.

Mothers were donors in 38%, fathers in 29% and siblings in 33% of transplants (Table 2). The median age of the donors was 34.7 years (range 16.3–47.7). The median number of CD34^+^ cells infused was 4.05 × 10^6^/kg (range 1.5–7.5). There was only one patient with B-ALL conditioned with BU-FLU-MEL and given 4.8 × 10^6^/kg CD34^+^ cells that developed primary graft failure but engrafted 11 days after a second conditioned PBSC transplant from the same donor. ANC of 0.5 x 10^9^/L was achieved at a median time of 16 days post-BMT (Figure 1A) while platelet engraftment occurred at a median of 30 days (Figure 1B). We found no correlation between the number of CD34^+^ × 10^6^/kg infused and time to neutrophil (*p* = *0.86, R*^2^ = 0.002) or platelet engraftment (*p* = 0.39, and *R*^2^ = 0.04). Mean absolute lymphocyte counts (ALC) doubled over time from 1.2 × 10^9^/L at 3 months to 2.4 × 10^9^/L at 1-year post haplo-BMT (Figure 1C). All patients, including the one receiving a second haplo-HCT, had complete donor chimerism on their day +28 bone marrows and as did all those that were re-assessed by peripheral blood chimerisms on days +100, +180, and +365.

### Graft-vs.-Host Disease

**Figure 2:**
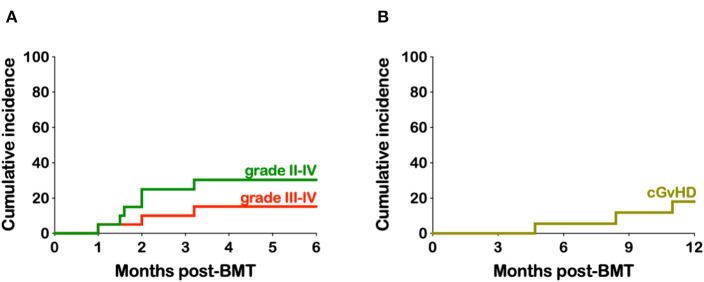
Kaplan-Meier estimates of acute and chronic- graft-vs.-host disease. **(A)** Cumulative incidence of grades II-IV and III-IV aGvHD. **(B)** Cumulative incidence of cGvHD.

The cumulative incidence of grades II to IV and III to IV acute GvHD (aGvHD) was 30.3 and 15.2% (Figure 2A). The three patients with grade III aGvHD all had stage III lower GI GvHD and responded to steroids. The cumulative incidence of chronic GvHD (cGvHD) and extensive cGvHD was 18.1 and 11.8%, respectively (Figure 2B). Of the two patients with extensive cGvHD one had GI, skin, and joint involvement while the other had GI, skin and liver. A third patient had cGvHD limited to his oral mucosa. All patients responded to steroid therapy and are all off immunosuppression with resolution of their cGvHD symptoms.

### Infections

All patients undergoing haplo-BMT have three lines, usually a double lumen broviac or double lumen PICC line and a port-a-cath. In the first 100 days post-BMT there were 14 blood cultures drawn from at least one lumen that grew Gram-positive bacteria in 12 patients (57%). All these occurred between day +0 and day +24 (Table 3). In 10 of the 12 patients (83%) the gram+ infections arose before neutrophil engraftment. All patients responded promptly to appropriate antibiotic therapy except for one who died from overwhelming methicillin resistant staphylococcus aureus (MRSA) sepsis which developed on day +10. Of note is that this patient had required prolonged ventilatory support for MRSA infection following reinduction chemotherapy months prior to her BMT. There were three Gram-negative line infections/bacteremias in two patients. One patient died from septic shock after developing recurrent bacteremia with multi-drug resistant Enterobacter cloacae. There were no fungal infections documented in any of our patients undergoing haplo-BMT.

**Table 3:** Central line infections/Bacteremias and fungal infections.

First 100 days post BMT *n* (%)*	
**Gram (+)**	**14 (57.1)**
Coagulase negative staphylococcus	5
*Staphylococcus aureus*	1
Streptococcus	3
Enterococcus	2
Other gram (+) bacteria	3
**Gram (–)**	**3 (9.5)**
Klebsiella	1
Enterobacter	2
**Fungal**	**0**

* *Total of 14 gram (+) positive cultures in 12 patients, total of 3 gram (**–**) positive cultures in two patients. CMV, cytomegalovirus*.

**Figure 3:**
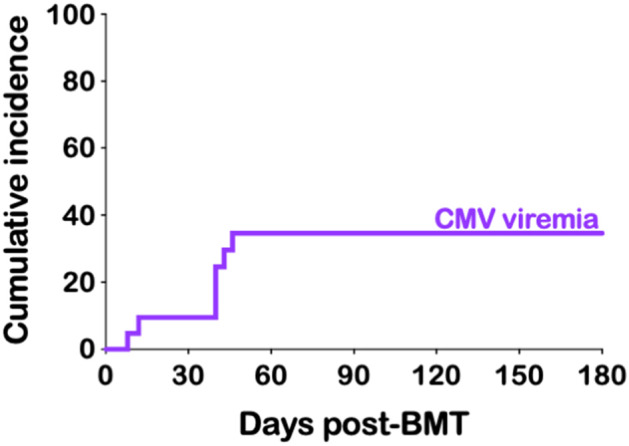
CMV viremia. Cumulative incidence of CMV viremia.

CMV reactivation was not uncommon after haplo-BMT. All of our 21 patients were at risk (seropositive recipient and/or seropositive donor) with seven developing CMV viremia (33%) (Table 4). The median time to peak CMV viremia was day +40 post haplo-BMT (Figure 3) with viral loads peaking between 700 and 11,500 IU/ml. All patients responded to ganciclovir/valganciclovir, generally requiring 3–4 weeks of anti-viral therapy. BK viruria (>7.5 × 10^8^ viral copies/ml) was detected in four patients (19%) with symptoms of BK hemorrhagic cystitis consisting of dysuria, frequency and microscopic or macroscopic hematuria (Table 4). A patient that developed end stage renal failure requiring dialysis also had significant BK viremia of 7500 viral copies/ml and was treated with cidofovir. None of our patients had clinically significant reactivation of EBV, HHV-6 or adenovirus warranting therapeutic intervention.

**Table 4:** CMV viremia and BK viruria.

**First 100 days post BMT** ***n*** **(%)**	
**CMV status**	
R+/D+	6/14 (42.9)
R+/D–	1/6 (16.7)
R–/D+	0/1 (0)
**Total at risk**	**7/21 (33.3)**
R–/D–	0/0 (0)
**BK virus**	
BK viruria >7.5 × 10^8^	4/21 (19)

*R, recipient; D, donor; CMV, cytomegalovirus*.

### Transplant Related Toxicities

**Figure 4:**
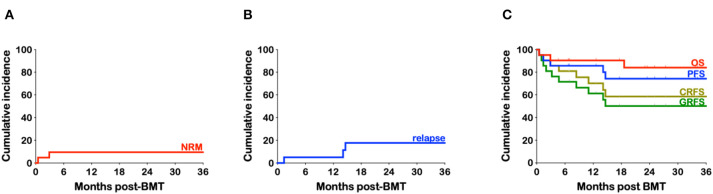
Kaplan-Meier estimates of non-relapse mortality, relapse, and survival. **(A)** Cumulative incidence of non-relapse mortality (NRM) **(B)** Cumulative incidence of relapse. **(C)** Cumulative incidence of overall survival (OS), progression-free survival (PFS), chronic graft-vs.-host-free relapse-free survival (CRFS) and grade III-IV aGvHD-free, cGvHD-free, relapse-free survival (GRFS).

Three patients (14.3%) required admission to the intensive care unit (ICU) within their first 100 days post-BMT (Table 5). Two of these patients required mechanical ventilation. One died MRSA sepsis as noted above (Figure 4A). The second patient who needed mechanical ventilation had developed significant ascites from veno-occlusive disease (VOD) compromising his respiratory effort and requiring CRRT for hepato-renal syndrome. This patient with infant leukemia had a short-lived response to CAR-T cell therapy and achieved a CR3 following inotuzumab ozogamicin known to predispose to VOD. As noted above, he eventually succumbed to septic shock following recurrent bacteremia with multi-drug resistant Enterobacter cloacae and was one of two patients contributing to a non-relapse mortality (NRM) of 9.5%. The third patient admitted to ICU did so for CRRT after developing renal failure from BK nephritis (as noted above) and cidofovir treatment and after prolonged therapy with liposomal amphotericin B prior to his haplo-BMT. He was also our sole patient that had failed engraftment and received a second PBSC transplant. He remains on renal dialysis.

**Table 5:** Transplant related toxicity.

**First 100 days post BMT** ***n*** **(%)**	
Admission to ICU	3 (14.3)
Mechanical ventilation	2 (9.5)
CRRT/dialysis	2 (9.5)
VOD/SOS	1 (4.8)
TRM	2 (9.5)

*BMT, bone marrow transplant; ICU, intensive care unit; CRRT, continuous renal replacement therapy; VOD/SOS, nonocclusive disease/sinusoidal obstruction syndrome; TRM, transplant related mortality*.

### Survival

Only three patients have relapsed, all which were heavily treated relapsed/refractory young adults (24, 23, and 24 years). One with HD, had failed autologous PBSC transplantation and was only in partial remission prior to haplo-BMT, another had refractory diffuse large B cell lymphoma (DLBCL) never having achieved a CR despite multiple lines of therapy, including CAR-T cells, and the third patient had B-ALL since she was 12 years of age with complex cytogenetics and had at least six prior relapses including one after an unrelated BMT. The cumulative incidence of relapse is 17.6% (Figure 4B). With a median follow-up of 25.1 months (range 4.6–52.9) the overall survival (OS) is 84%, with a progression-free survival (PFS) at 74.3% (Figure 4C). Taking into consideration both relapse and grade III-IV acute or chronic GvHD requiring treatment, the GvHD-free relapse-free survival (GRFS) at 2 years stands at 50.1% while the cGvHD-free, relapse-free survival (CRFS) at 58.5% (Figure 4C).

## Discussion

The preferred donor for allogeneic HCT is an HLA-matched sibling. However, <30% of patients will have a matched sibling donor (MSD), a probability that continues to decline in developed countries due to decreasing birth rates. Notably, the likelihood of having an MSD is estimated to be only 22% for the U.S. pediatric population (0–19 years) and is even lower in younger patients (1–5 years) at 17% (18). Traditionally, seeking a matched unrelated donor (MUD) is considered the second-best alternative after an MSD. While national and international hematopoietic cell registries have diversified and expanded in an attempt to increase access to unrelated donors, finding a MUD has continued to be a challenge for minority populations. Younger pediatric patients who do not have an MSD may have the option of receiving umbilical cord blood transplant (UCB) in place of a MUD. As UCB units are cryopreserved, they are readily available. The low numbers of T cells in UCB allows for mismatched units to be utilized, thereby expanding the donor pool for younger pediatric patients. However, disadvantages of UCB include low numbers of hematopoietic stem cells, which are associated with slow engraftment, and the high cost of cord blood unit acquisition. The current trend in the U.S. and especially Europe now favors the use of haplo-HCT over UCB transplants, particularly for malignant diseases (19, 20).

The benefits of haploidentical over unrelated donor (URD) HCT are numerous, with arguably the most notable being that haplo-HCT extends donor availability to nearly all patients. With more than half of our patients being ethnic and/or racial minorities (Table 2), our program relies heavily on haploidentical familial donors as an unrelated donor is secured in <40% of our patients (21). In fact, since the initiation of our pediatric haplo-BMT program in October 2015 we have performed 3-fold more haplo-BMTs than URD HCTs for hematologic malignancies. Haplo-BMT offers additional advantages by circumventing the delays and costs associated with unrelated donor searches and hematopoietic stem cell procurement. Haplo-HCT, therefore, can expedite transplantation in time-sensitive circumstances such as pediatric acute leukemias potentially preventing relapses. Moreover, haploidentical familial donors, especially parents, which were donors in two-thirds of our patients, are eager to donate and readily available not only for the initial harvest but also potential additional collections of bone marrow, PBSCs or donor leukocyte infusions (DLI), if needed.

Early attempts at T-cell depleted haplo-HCT in the 1980s proved to be challenging for various reasons, including a high incidence of graft rejection and delayed immune reconstitution leading to infections and relapse (22, 23). After decades of research a more refined graft engineering approach to T-cell depletion used primarily in Europe consists of haplo-HCT with αβ T and B cell depleted grafts. This transplant methodology allows for the transfer of CD34^+^ stem cells, without GvHD inducing αβ T cells, but with inclusion of and NK and γδ T-cells, both of which are capable of eliciting anti-leukemic and anti-pathogenic effects. A group from Germany reported their results with this approach in pediatric patients with mostly advanced hematologic malignancies (24). A team from Italy more recently added their experience in acute leukemia pediatric patients in complete remission (25). Patients received MAC which was TBI based in the majority of cases. Primary graft failure was low, there was an absence of grade III-IV aGvHD, with only 5% cGvHD. With a median follow-up of 46 months OS, PFS and relapse were 72, 71, and 24% respectively. Comparison of their haplo-HCT outcomes was performed to their acute leukemia patients in CR that received MSD or MUD HCT during the same time period, with all three populations having comparable disease characteristics. A lower incidence of grade III-IV aGvHD and cGvHD was observed in haplo-HCT patients with no significant difference in PFS amongst the three transplant groups.

Fifty-seven years ago, it was found that a single dose of CY, if given between the first and fourth day following implantation of a skin graft from a haploidentical donor, it was able to prolong the survival of the allograft (26). In the setting of T-cell replete hematopoietic grafts, GvHD prevention becomes of utmost importance, given that the graft contains all of the immune cells necessary to attack the immunocompromised host. Therefore, pre- and especially post-transplant immunosuppression are essential. The use of PT-CY originated at Johns Hopkins University in experimental murine models of HCT and was successfully translated clinically (27). PT-CY effectively targets rapidly dividing alloreactive donor T-cells responsible for GvHD while not affecting quiescent hematopoietic stem cells which express high levels of aldehyde dehydrogenase (28, 29). Moreover, this approach is simple as it circumvents the need to *ex-vivo* manipulate stem cell grafts and thus can be applied by almost any center performing allogeneic HCT.

There have been no randomized trials comparing haploidentical to MUD HCT. Such trials are difficult to conduct given the diverse disease conditions, conditioning regimens, donor characteristics, stem cell sources and GvHD prophylaxis utilized. However, countless adult haplo-HCT trials for hematologic malignancies have been conducted generally reporting comparable outcomes to concurrently reported MUD HCT (2, 3, 5, 30). Many of these contemporaneous studies have indicated that haplo-HCT may be associated with less acute and chronic GvHD with no differences in NRM, relapse and OS compared to MUD-HCT. Similarly, there have been no randomized studies directly comparing mismatched unrelated donor (MMUD) and haplo-HCT, but most reports indicate that outcomes with MMUD-HCT are inferior to haplo-HCT (31–36). This has led many centers, including ours, to favor the selection of a haploidentical donor over a MMUD. There has been however, a Phase III, randomized trial of RIC comparing double unrelated umbilical cord blood (dUCB) to haplo-BMT (BMT-CTN 1101) the results of which have not been published yet.

Despite the numerous adult reports there have only being a limited number of pediatric studies utilizing PT-CY in patients with hematologic malignancies. As shown in Table 1, the two largest studies used primarily or exclusively RIC (6, 8). While it is difficult to compare outcomes from these reports given the generally advanced stages of disease including >CR2 and, in many cases, refractory disease, PFS trended lower in RIC patients compared to MAC underscoring the consideration of more intensive regimens for better disease control. Two Asian studies have reported on the use of chemotherapy based MAC followed by T-cell replete PBSC transplant and PT-CY (7, 10). There were no graft failures, severe acute and chronic GvHD were low and improved PFS correlated with CR status. A recent conference abstract from nine US and Canadian institutions using myeloablative BU-CY or TBI-CY, surprisingly reported high graft failure and relapse rates at 1-year despite their patients being in CR at time of BMT (Table 1) (11).

In our updated report of 21 pediatric and young adult patients we have continued to observe excellent results with our MAC regimens. For patients with B-ALL we primarily use a TBI-based regimen previously described in adult patients by the Genoa group (2). For non-ALL patients we have been applying a chemotherapy-based MAC regimen comprised of BU-FLU-MEL. Coincidently, our BU-FLU-MEL regimen resembles that reported by Jaiswal et al. with regards to busulfan, but ours has 4 days of FLU rather than five which is infused following BU rather than concurrently, and includes a lower dose of MEL (100 mg/m^2^ instead of 140) (7, 9, 12). While the majority of our patients received PT-CY, six patients were part of our phase I study which is progressively substituting PT-BEN for PT-CY. This trial is based on our murine investigations demonstrating that PT-BEN has advantages in preserving GvL effects over PT-CY (37). While all six patients received PT-CY on day +3, three patients had partial substitution and three complete substitution of PT-CY with PT-BEN on day +4. A report of our preliminary trial findings is in press (38).

Our outcomes remain excellent, with donor engraftment occurring in >95% of patients. We observed a high incidence of mostly Gram positive bacterial infections but no documented fungal infections. The incidence of CMV viremias and BK virurias was similar to other haplo-HCT reports (1, 4, 39–44). The cumulative incidence of grades III-IV aGvHD and cGvHD were low at 15.2 and 18.1%, respectively, with all patients responding to steroid therapy. With a median follow-up of 25.1 months the OS and PFS at 2 years is 84 and 74.3% which compares favorably to the published pediatric haplo-HCT studies (6–8, 10, 11, 24, 25). As noted above the only three patients relapsing after haplo-BMT all had advanced disease with two not in remission at time of BMT, two had failed previous transplants and one had not responded to CAR-T cell therapy. CRFS, which is a composite end point of survival without cGvHD requiring systemic therapy or relapse and GRFS, the endpoint of grade III-IV aGVHD or systemic therapy–requiring cGVHD or relapse are 58.5 and 50.1%, respectively at 2-years. Previous, pediatric T-cell replete pediatric haplo-HCTs have not reported on this composite end point. However, Locatelli's group compared outcomes in a younger pediatric population (<18 years) with acute leukemia in remission receiving haplo-HCT with αβ T and B cell depleted grafts, MUD or MMUD and reported 5-year PFS of 62, 65, and 55% and CRFS of 58, 67, and 34% (45) which parallels our 2-year haplo-BMT PFS of 74.3%, and CRFS of 58.5%. Moreover, our GRFS and CRFS with haplo-BMT appear superior when compared to a recent CIBMTR analysis of over 1,600 pediatric acute leukemia patients receiving either a UCB or a MMUD transplanted in CR that found GRFS of 33 and 22% and CRFS of 38 and 27%, respectively (46). We realize that our numbers are small, but to provide a contemporaneous comparison between haploidentical and MUD transplantation, we analyzed the outcomes of our patients undergoing MUD transplantation for hematologic malignancies performed on our unit during the same period. The 2-year OS, PFS, CRFS and GRFS of our patients receiving MUD transplantation is 71.5, 71.5, 42.9, and 42.9% compared to 84, 74.3, 50.1, 58.5% in those receiving haplo-BM.

In summary, the use of haplo-HCT has advanced faster in adult HCT programs compared to pediatric and in Europe and Asia compared to North America. Various options exist with respect to the choice of conditioning regimen, graft manipulation and GvHD prophylaxis. The introduction of PT-CY has transformed haplo-HCT into a practical procedure easily applicable in every transplant center. Our experience continues to support the application of MAC T-cell replete haplo-BMT as a safe and effective alternative to MUD HCT.

## Data Availability Statement

The datasets generated for this study are available on request to the corresponding author.

## Ethics Statement

The studies involving human participants were reviewed and approved by Human Subjects Protection Program The University of Arizona. Written informed consent to participate in this study was provided by the participants' legal guardian/next of kin.

## Author Contributions

EK reviewed and analyzed the data and wrote the manuscript. He also designed and is PI of the clinical trial. LS collected and reviewed data and edited the manuscript. SR, NR, and BS reviewed the data and edited the manuscript. All authors contributed in the treatment of the patients.

## Conflict of Interest

The authors declare that the research was conducted in the absence of any commercial or financial relationships that could be construed as a potential conflict of interest.
